# *Dendrobium officinale polysaccharide* Converts M2 into M1 Subtype Macrophage Polarization via the STAT6/PPAR-r and JAGGED1/NOTCH1 Signaling Pathways to Inhibit Gastric Cancer

**DOI:** 10.3390/molecules28207062

**Published:** 2023-10-12

**Authors:** Yi Zhao, Xuefeng Lu, Hongxia Huang, Yao Yao, Hongning Liu, Youzhi Sun

**Affiliations:** 1 Research Center for Differentiation and Development of Basic Theory of Traditional Chinese Medicine, Jiangxi University of Chinese Medicine, Nanchang 330004, China; 20070987@jxutcm.edu.cn (Y.Z.); ispf22y@163.com (X.L.); 18216180262@163.com (H.H.); yaoyaoyy0917@163.com (Y.Y.); 2 School of Chinese Medicine, Jiangxi University of Chinese Medicine, Nanchang 330004, China

**Keywords:** *Dendrobium officinale polysaccharide* (DOP), macrophage polarization, STAT6/PPAR-r signaling pathway, JAGGED1/NOTCH1 signaling pathway, gastric cancer

## Abstract

*Dendrobium officinale polysaccharide* (DOP) has shown various biological activities. However, the ability of DOP to participate in immune regulation during anti-gastric cancer treatment has remained unclear. In this study, the in vitro results showed that DOP has the potential to polarize THP-1 macrophages from the M2 to the M1 phenotype, downregulate the STAT6/PPAR-r signaling pathway and the protein expression of their down-targeted ARG1 and TGM2, and further decrease the main protein and mRNA expression in the JAGGED1/NOTCH1 signaling pathway. DOP suppressed the migration of gastric cancer cells by decreasing the protein expression of N-cadherin and Vimentin and increasing E-cadherin. In addition, CM-DOP promoted the apoptosis of gastric cancer cells by upregulating Caspase-3 and increasing the ratio of Bax/Bcl-2. In vivo, DOP effectively inhibited the growth of tumors and the expression of Ki-67. In summary, these findings demonstrated that DOP converted the polarization of M2 subtype macrophages into M1 subtypes via the STAT6/PPAR-r and JAGGED1/NOTCH1 signaling pathways in order to reduce apoptosis and prevent migration, thus indicating the potential of DOP as an adjuvant tumor therapy in preclinical and clinical trials.

## 1. Introduction

**Figure 1:**
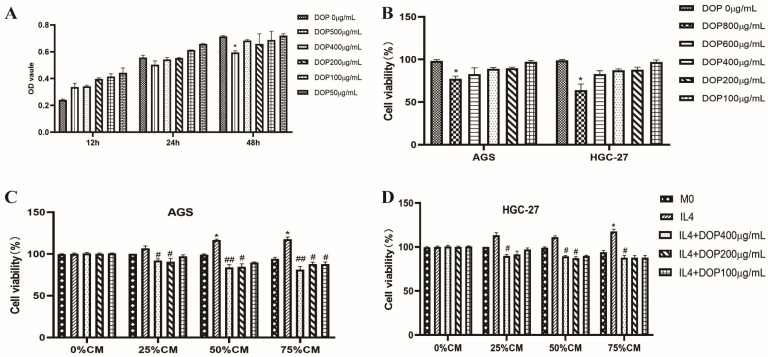
Effect of DOP and macrophage-conditioned medium on THP-1 and gastric cancer cell proliferation. (**A**) Effect of DOP on THP-1 cell proliferation. (**B**) Effect of DOP on gastric cancer cells. (**C**,**D**) Effect of DOP-treated macrophage-conditioned medium on gastric cancer cells. * *p* < 0.05 vs. non-treatment group or M0 group, respectively; # *p* < 0.05, ## *p* < 0.01 vs. IL-4-treated group (M2 group). Data are expressed as mean ± SD, N = 3, and tests were repeated three times.

**Figure 2:**
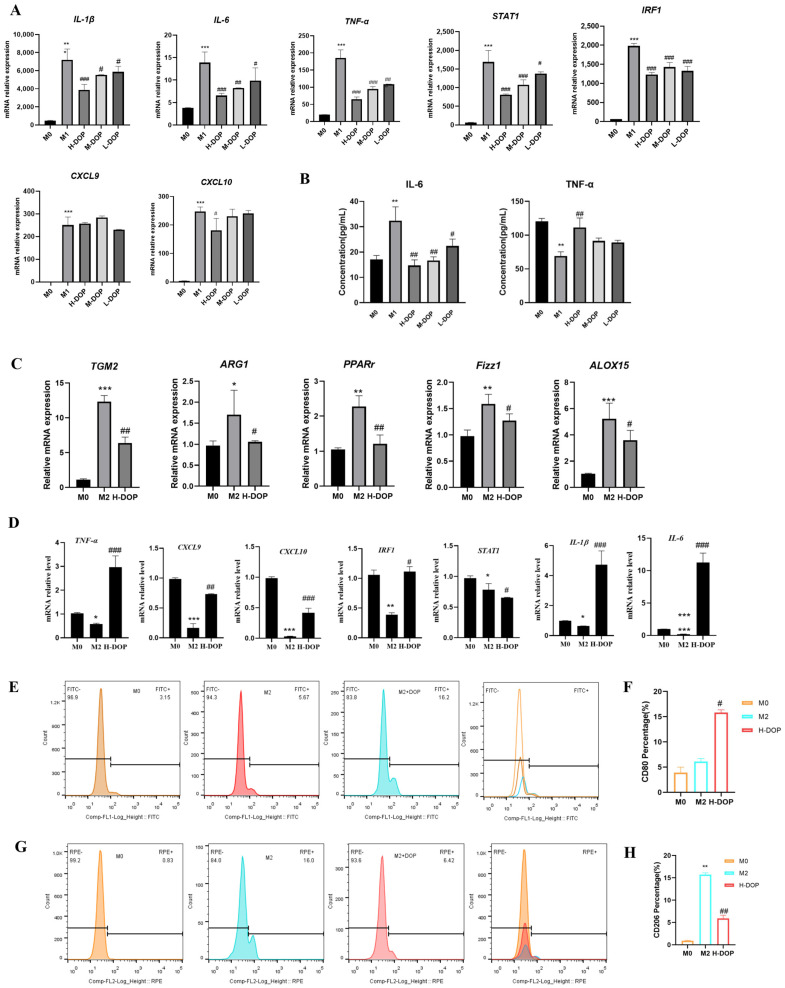
DOP promoted the expression of genes associated with the M1-like phenotype and induced the translation of M2-like macrophages into the M1-like phenotype. (**A**) THP-1 cells were exposed to IFN-γ (100 ng/mL) and LPS (100 ng/mL) for 24 h, which induced M1-like macrophages, and then treated with or without DOP (100, 200, 400 μg/mL) for a further 4 h. (**B**) The secretion of TNF-α and IL-6 were detected via ELISA assay. (**C**) The mRNA expression of M2-type macrophage markers CD206, CD163, Arg1, IL-10, Fizz1, Alox15, and PPARr in THP-1 cells under IL-4 stimulation for 24 h, treated with or without DOP (200 μg/mL) for another 24 h. (**D**) The mRNA expression of M1-type macrophage markers IL-6, CXCL9, CXCL10, IRF1, TNF-α, STAT1, and IL-1β in M2-type macrophages (**E**–**H**). DOP inhibits the expression of CD206 and upregulates the expression of CD80 in M2-type macrophages, as shown via flow cytometry. N = 3, and tests were repeated three times, Data are expressed as mean ± SD, * *p* < 0.05, ** *p* < 0.01 and *** *p* < 0.001 vs. M0 group; # *p* < 0.05, ## *p* < 0.01 and ### *p* < 0.001 vs. M2 group.

Gastric cancer is still one of the most common malignant tumors; its incidence and mortality are ranked as fifth and fourth, respectively, among global cancer figures [1,2]. With the arrival of the aging era, it is expected that the incidence of cancer will continue to increase in the high-incidence regions, which could increase the cancer burden and cause considerable pressure on the current Chinese medical and health service system [3,4]. Patients are frequently diagnosed in the late stage of GC, which misses the critical treatment period [5]. It has been reported that immunotherapy has improved traditional chemotherapy and ushered in a new era in cancer treatment [6]. However, it is unfortunate that up to 70% of ICI-treated patients do not respond to immune checkpoint inhibitors (ICIs) [7]. 

Macrophages are an integral part of innate immunity. Due to their plasticity, they are widely used in the study and treatment of many diseases. M1 macrophages exert both anti-inflammatory and anti-tumorigenic functions, triggered by pro-inflammatory cytokines, while M2 macrophages present anti-inflammatory and tumor-promoting capabilities, which are stimulated by Th2 cytokines. Tumor-associated macrophages (TAMs) commonly appear in advanced tumors and show the characteristics of the M2 phenotype, which strongly affect the level of cytokines and metabolites in the tumor microenvironment [8]. Therefore, understanding the regulatory factors and mechanisms of macrophage polarization in tumor microenvironments (TMEs) will be helpful in discovering drugs targeting M2 macrophages. A number of important and frequently employed pathways in macrophage polarization have been reported; for example, peroxisome proliferator activating receptors (PPAR-gamma), signal transducers, activators of transcription (STATs), etc. [9,10]. 

It has been reported that the NOTCH signaling pathway is a key factor in the differentiation process of tumor-associated macrophages (TAMs) [11,12]. When cancer cells exhibit a high expression of JAGGED1 and increase TAM markers in macrophages, after treatment with g-secretase inhibitors which can block NOTCH signaling, the expression of JAGGED1 is decreased [13]. Moreover, cytokine IL-4 can activate the NOTCH signaling pathway and increase the expression of JAGGED1 and PPAR [14]. Zhao’s research found that the activation of the NOTCH pathway in macrophages hinders tumor growth via the amplification of the miR-125a expression and the deactivation of TAMs [15]. Therefore, the inhibition of the JAGGED1/NOTCH1 signaling pathway may be a useful way to inhibit M2 macrophages. 

Natural polysaccharides (NPs) have various regulatory effects on macrophages, such as influencing the phenotype of macrophages [16]. Over the years, attention has been paid to the role of polysaccharides in resetting the phenotype of M2-like macrophages [17]. NPs have demonstrated anti-GC activity in cellular and animal studies, as well as clinical trials [18]. Dendrobium officinale belongs to the orchid family. It can regulate immunity and improve gastrointestinal function after long-term use, including in cases of digestive disorders, gastric ulcers, gastric mucosal injury, gastritis, and gastric cancer [19,20,21]. D. officinale polysaccharide (DOP) is used as the main active constituent [22,23,24]. Zhang previously reported that the inhibitory effect of DOP on gastric cancer SGC-7901 xenograft tumors in nude mice may be attributed to an elevation in TNF-a and IL-2 levels, as well as the upregulation of Bax and the downregulation of the Bcl-2 protein expression [25]. Moreover, Wang reported that DOP inhibited tumor growth in MFC, CT26, and LLC cells, with a mechanism via direct regulation of the expression of TLR2 in TAMs and the promotion of its expression in the M1 phenotype [26]. To date, there have been no reports on whether DOP inhibits gastric cancer by regulating macrophage polarization and the associated mechanism involving the JAGGED1/NOTCH1 signaling pathway. Thus, we explored the anti-tumor effect of DOP on gastric cancer cells by converting M2 into M1 subtype macrophage polarization via STAT6/PPAR-r and JAGGED1/NOTCH1 signaling pathways, as a result, DOP reduces apoptosis and prevents migration in gastric cancer. 

## 2. Results

### 2.1. Effect of DOP and Macrophage-Conditioned Medium on THP-1 and Gastric Cancer Cell Proliferation 

After the M0 macrophages were induced via PMA for 24 h, the CCK-8 method was used to detect the effects of different concentrations (50–500 μg/mL) of DOP on the proliferation and inhibition of M0 macrophages after 24 h and 48 h. Figure 1A shows that the DOP treatment at a concentration below 500 μg/mL exerted no significant growth inhibition on the cell line. However, based on the cell morphology, a high concentration (500 μg/mL) caused cell deformation, changing the shape of the cells from a round to a long spindle. Therefore, it was not recommended to use a DOP concentration higher than 500 μg/mL for the subsequent experiments. Furthermore, Figure 1B shows the direct inhibitory effect of DOP on HGC27 and AGS gastric cancer cells; a low concentration of DOP (100, 200, or 400 μg/mL) exhibited no toxicity on HGC27 and AGS gastric cancer cells, but for a concentration up to 800 μg/mL, the cell viability of HGC27 and AGS cells was lower than that of the control group, and this difference was statistically significant (*p* < 0.05, *p* < 0.05, respectively).

The cytotoxic effect of different proportions (25%, 50%, and 75%) of the M2-like macrophage-conditioned medium on HGC27 and AGS cells was investigated via a CCK-8 assay. Figure 1C,D indicates that the different proportions of the conditioned medium of(CM) from the M2-like macrophages could promote the viability of cells, particularly the 75% conditioned medium, which significantly promoted cell growth. In contrast, the CM from the DOP-treated M2-like macrophages significantly inhibited the cell viability of the two gastric cancer cell lines, which was comparable to the effect of the CM from the untreated M2-like macrophages. 

### 2.2. Effect of DOP on M1 and M2 Subtype Macrophages

THP-1 cells were induced via PMA for 24 h and then sequentially differentiated into M0 macrophages. The M1 macrophages were induced via IFN-γ and LPS for 24 h and treated with DOP for 24 h. Figure 2A displays the M1 genes IL-6, CXCL9, CXCL10, IRF1, TNF-α, STAT1, and IL-1β that were detected via RT-PCR, which all decreased after treatment with the three concentrations of DOP. Figure 2B shows that the DOP treatment led to a markedly reduced secretion of TNF-α and IL-6, which are typical proinflammatory cytokines in the supernatant of M1-like macrophages, as detected via the ELISA assay.

In order to confirm the effect of DOP on the M2-like polarization, M2 marker genes were evaluated for specificity via RT-PCR. After treatment with a 400 μg/mL concentration of DOP for 24 h, the mRNA levels of Arg1, TGM2, Fizz1, Alox15, and PPARr, which were used as M2 marker genes, were all decreased, exhibiting statistically significant reductions when compared to the M2-like macrophage group (*p* < 0.05, *p* < 0.05, *p* < 0.05, *p* < 0.05, *p* < 0.01, *p* < 0.05, respectively; Figure 2C).

To verify the effect of DOP on the conversion of M2-like macrophages into M1-like macrophages, we evaluated the M2-like specific marker genes using RT-PCR. The M1 marker genes IL-6, CXCL9, CXCL10, IRF1, TNF-α, STAT1, and IL-1β were assessed via RT-PCR in M2-like macrophages after being treated with DOP for 12 h. The data showed that the mRNA expression levels of IL-6, CXCL9, CXCL10, TNF-α, and IL-1β were upregulated in the M2-like macrophages, exhibiting statistically significant results (*p* < 0.01, *p* < 0.01, *p* < 0.001, *p* < 0.01, *p* < 0.01, respectively; Figure 2D). Figure 2E,F shows that, compared with the M0 group, the expression of CD206, a characteristic M2 surface molecule, was significantly increased in IL-4-induced THP-1 cells, reaching 16.0%. After treatment with DOP, this increase was reduced to 6.42%, which indicates that DOP decreased the M2 subtype macrophages. Moreover, Figure 2G,H shows that the surface marker of M1 macrophages expressing CD80 was decreased in the M2 subtype macrophages, and the proportion of CD80 decreased by 5.67%. After treatment with DOP, the proportion of CD80 increased by 16.2%, indicating that DOP promoted the surface marker expression of the M1 subtype in M2 subtype macrophages, leading to a transition into M1 subtype macrophages. As shown via flow cytometry, DOP effectively inhibited the IL-14-induced M2-like polarization of macrophages and translated them into M1 macrophages.

### 2.3. Effect on Transcription Factors in the STAT6/PPAR-r Pathway and its Down-Targeted Proteins in M2-like Subtype Macrophages

**Figure 3:**
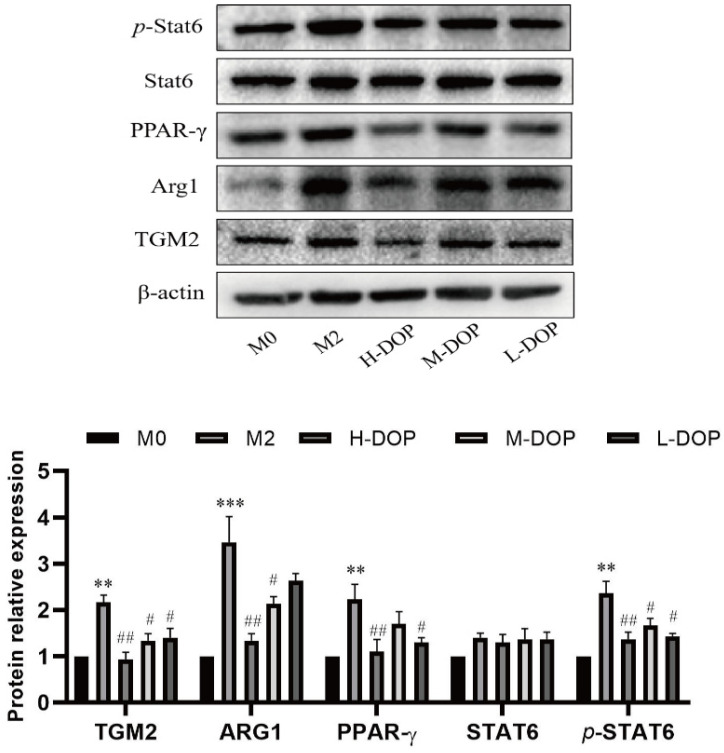
Effect of DOP on transcription factors STAT6/PPAR-r and down-targeted proteins in M2-like macrophages. ** *p* < 0.01 and *** *p* < 0.001 vs. M0 group; # *p* < 0.05, ## *p* < 0.01 vs. M2 group. N = 3, and tests were repeated three times. Data are expressed as mean ± SD.

Figure 3 illustrates that there was a statistically significant increase in the protein expression of *p*-STAT6 and PPAR-r in the M2-like macrophage group, leading to the upregulation of the ARG1 and TGM2 expression compared to the M0 group (*p* < 0.01, *p* < 0.01, *p* < 0.001, and *p* < 0.01). After treatment, the protein expression levels of p-STAT6, PPAR-r, ARG1, and TGM2 were all decreased. These data indicate that DOP impeded the differentiation and expression of macrophages with M2-like characteristics.

### 2.4. DOP-Inhibited JAGGED1/NOTCH1 Signaling Pathway in M2-like Macrophages

**Figure 4:**
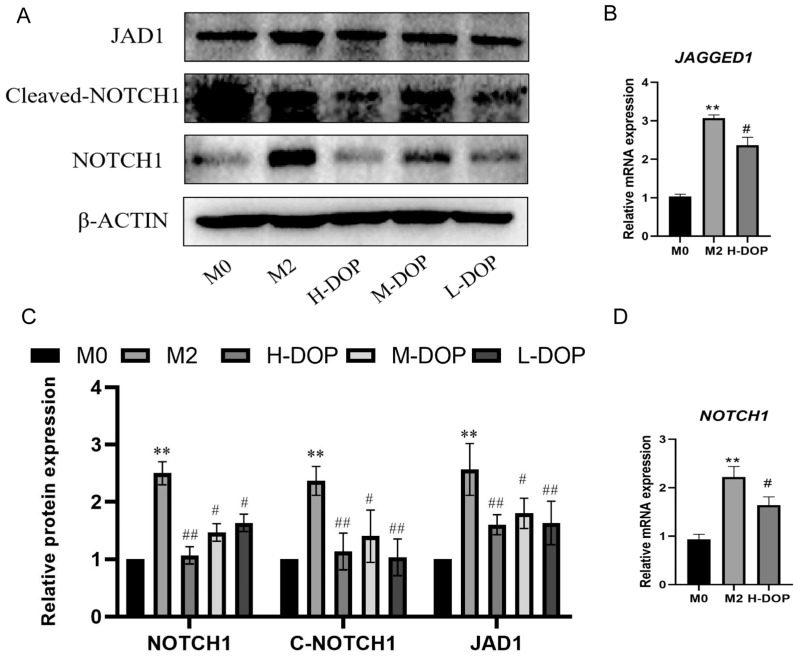
DOP activated JAGGED1/NOTCH1 signaling pathway in M2-like macrophages. (**A**,**C**). Western blotting analysis (**A**) and quantification of data (**C**) of JAGGED1, NOTCH1, and Cleaved-NOTCH1 in THP-1 cells co-treated with or without DOP under IL-4 stimulation for 24 h. (**B**,**D**) mRNA expression of *JAGGED1* and *NOTCH1*. N = 3, and tests were repeated three times. Data are expressed as mean ± SD, ** *p* < 0.01 vs. M0 group; # *p* < 0.05, ## *p* < 0.01 vs. M2 group.

The effect of DOP on the JAGGED1/NOTCH1 signaling pathway via the activation of M2-like macrophages was assessed. When compared with the M0 macrophages, the M2-like macrophages presented an increased protein expression of *JAGGED1, NOTCH1*, and Cleaved-*NOTCH1*, as shown in Figure 4 (*p* < 0.01). After the DOP treatment, the expression levels of *JAGGED1,* NOTCH1, and Cleaved-NOTCH1 were significantly downregulated. Simultaneously, the DOP treatment inhibited the mRNA expression of *JAGGED1* and *NOTCH1*, as shown via RT-PCR. These results indicate that DOP inhibited the JAGGED1/NOTCH1 signaling pathway, thus inhibiting the expression of M2-like macrophages.

### 2.5. Effect of CM-DOP on Promoting Apoptosis and Inhibiting Migration in Gastric Cancer Cell

**Figure 5:**
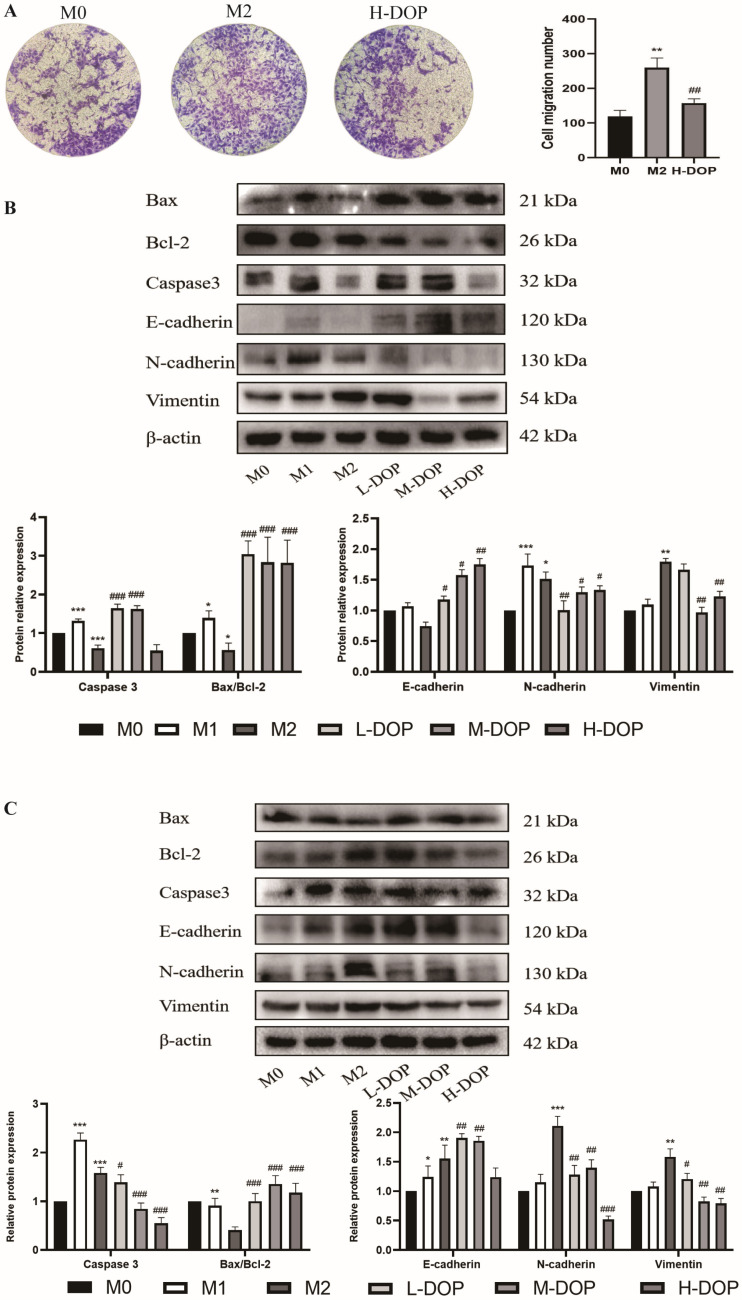
The effect of the CM-DOP on the migration and apoptosis of gastric cancer cells. (**A**) CM-DOP inhibited the migration of HGC-27 cancer cells via Transwell chamber. (**B**,**C**) CM-DOP inhibited the migration and promoted apoptosis in AGS (**B**) and HGC-27 (**C**) gastric cancer cells. N = 3, and tests were repeated three times. Data are expressed as mean ± SD, * *p* < 0.05, ** *p* < 0.01 and *** *p* < 0.001vs. M0 group; # *p* < 0.05, ## *p* < 0.01 and ### *p* < 0.001vs. M2 group.

The conditioned medium (CM) of TAMs was then used to study the effects of TAMs on gastric cancer cell proliferation, migration, and invasion. To investigate the effects of DOP on M2-like macrophages in inhibiting the migration of HGC-27 cells, we used a Transwell chamber. Figure 5A shows that, compared with CM-M0, CM-M2 could significantly promote the migration of HGC-27 cells, and this difference was found to be statistically significant (*p* < 0.01). CM-H-DOP could significantly inhibit the number of migrating cells with statistical significance (*p* < 0.01).

The Western blot results showed that CM-M2 was able to downregulate the expression of Caspase-3 in AGS and HGC-27 cancer cells, as well as reduce the ratio of Bax/Bcl-2 in the three gastric cancer cells, whereas the CM-DOP treatment significantly upregulated Caspase-3 and increased the ratio of Bax/Bcl-2. Moreover, CM-M2 promoted the N-cadherin protein expression in HGC-27 cancer cells. However, the CM-DOP treatment reversed the effects of N-cadherin and Vimentin, as well as increased the E-cadherin expression in AGS and HGC-27 cancer cells, especially in H-DOP (Figure 5B). These data indicate that CM-DOP showed an ability to promote apoptosis and inhibit gastric cancer cells.

### 2.6. Effect of DOP on Gastric-Xenografted Tumor Model In Vivo 

**Figure 6:**
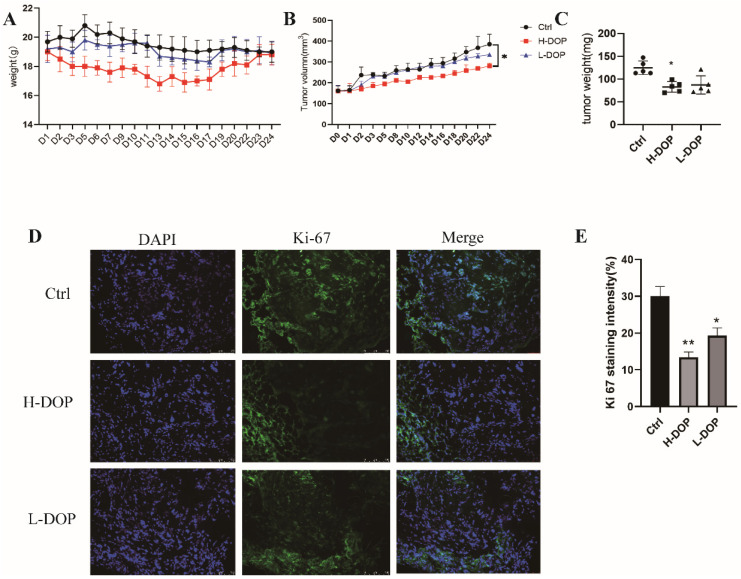
Effect of DOP on gastric-xenografted tumor model in vivo. (**A**) The change in mice weight. (**B**) Tumor volume. (**C**) Tumor weight. (**D**) Expression of Ki67 in tumor via fluorescent immunohistochemistry (IF). (**E**) The analysis of IF. N = 6, and tests were repeated three times. Data are expressed as means ± SD, * *p* < 0.05, ** *p* < 0.01 vs. control group.

We further established a gastric-xenografted tumor model to validate the anti-cancer effect of DOP. DOP had no obvious toxicity, which was demonstrated by the fact that the body weights of mice in all groups did not show significant changes during the treatment period. Moreover, the tumor volume and weight of H-DOP were reduced, and this difference was significant compared with that of the Ctrl group (non-treatment group; *p* < 0.05; Figure 6A–C), indicating that H-DOP significantly inhibited tumor growth. Furthermore, the Ki67 staining of tumor tissue sections from a subcutaneous gastric mouse model showed that DOP reduced the expression of Ki67 by approximately 50% (*p* < 0.01; *p* < 0.05; Figure 6D,E). 

## 3. Discussion

Tumor progression can result from an increased proportion of M2-like TAMs containing CD68+ and CD163+ [27]. In addition, M2-polarized TAMs can secrete TGF-β; thus, the present study investigated the anti-tumor efficacy of DOP in inhibiting M2 polarization, converting M2 into M1 subtype macrophages in gastric cancer cells in vitro and suppressing gastric cancer tumor growth in vivo. Importantly, the underlying anti-tumor mechanism of DOP was shown to inhibit the transduction of STAT6/PPAR-r signaling by targeting the STAT6 and PPAR-r proteins and the activation of the JAGGED1/NOTCH1 pathway, which may lead to the inhibition of M2 polarization, thereby significantly promoting apoptosis through upregulating Caspase-3, increasing the ratio of Bax/Bcl-2, and inhibiting cell migration by reversing the effects of N-cadherin and vimentin while also increasing the E-cadherin expression. These data suggest that DOP promotes the polarization of TAM from the M2-like phenotype to the M1-like phenotype macrophages through regulating and altering TAE, and thus, can be used as an effective drug to inhibit the development of gastric cancer.

It is commonly accepted that TAMs play a critical role in the progression of malignancies, including tumor proliferation, differentiation, stem cell (GSC) maintenance, angiogenesis, migration, and metastasis [28]. Moreover, the TAM phenotype is more likely to be an M2 type. Additionally, reprogrammed TAMs could be used as immunomodulatory therapies to inhibit tumor progression [29]. In clinical treatment, it has been proven that the combination of TAM-targeted therapy and immunosuppression is a more effective anticancer strategy [30,31]. Many studies have shown that DOP can interfere with the immune microenvironment by modulating macrophage polarization, thus inhibiting tumor growth [24,32]. For example, DOP suppresses the development of colorectal cancer (CRC) induced via AOM/DSS through augmenting the metabolic function of tumor-infiltrating CD8(+) cytotoxic T-lymphocytes (CTLs) and downregulating the expression of PD-1 in the CTLs, thereby bolstering the anti-tumor immune response [33]. DOPs have been successful in mitigating UPEC-promoted pyroptosis in macrophage cells. The mechanism involved inhibiting the NLRP3/Caspase-1/GSDMD pathway and activating ROS signals [34]. In the present study, we used an IL-4-induced macrophage M2 polarization model to investigate the potential influence of DOP on M1 and M2 macrophage polarization. The results of our RT-PCR analysis confirmed that DOP significantly inhibited the release of pro-inflammatory cytokines while simultaneously augmenting the anti-inflammatory cytokines in the M2 macrophage phenotype. Moreover, flow cytometry confirmed that DOP also efficiently inhibited the expression of CD206, a marker of M2 macrophages, and enhanced the expression of CD80, a marker of M1 macrophages, in M2 macrophages.

Transcription factors STAT6, IRF4, and PPARγ can regulate M2-type macrophage activation [35,36]. Activated STAT6 can relocate to the nucleus and bind directly to the promoters of certain downstream genes, including Fizzl. It further curbs the activation of NF-κ B, thus obstructing the transcription of some M1-related genes [35,37]. Previous reports have shown that AS1517499, a STAT6 inhibitor, has the ability to hinder M2 polarization in vitro, which subsequently leads to a decrease in the tumor growth rates and metastasis in a 4T1 mammary carcinoma model [38]. The activation of PPAR-γ can regulate the expression of anti-inflammatory genes, such as Arg1 and IL-10, causing macrophages to polarize toward the M2 phenotype [39]. The delayed recovery of the PPARγ expression and activity to resolve acute inflammation was observed following the inhibition of STAT6 activation [40]. For example, the process of pulmonary fibrosis was promoted by inducing M2-typed polarization via the activation of the STAT-6/KLF-4/PPAR-γ signaling pathway [41]. The suppression of STAT6 phosphorylation inhibited PPAR-γ molecules that were mediated via IL-4 in HMGECs [42]. In our study, DOP decreased the protein expression of p-STAT6 and PPAR-r, resulting in the downregulation of ARG1 and TGM2 in the M2-like macrophage group, which indicates that DOP hindered the differentiation of M2 macrophages by promoting the transcriptional activation of the PPAR-γ-related anti-inflammatory system in a STAT6-dependent manner. 

The NOTCH pathway, which has been shown to be highly active in the TME, is essential for the differentiation of M1 and M2 macrophages [43,44]. Previous studies have shown that the activation of NOTCH suppresses SIRP via HES1, which biases mouse macrophages toward M1 [45,46]. Recent reports have pointed out that NOTCH signaling was also activated after stimulation with IL-4 in the macrophages derived from human monocytes [14]. IL-4/IL-13-activated NOTCH1 affected the expression of the extracellular matrix, which caused macrophage polarization and osteogenesis in a mouse model [47]. In summary, the NOTCH pathway is considered to be an interesting target for multiple anti-tumor therapeutic strategies [48]. In our study, DOP intervention downregulated the protein expression of Jad1, NOTCH1, and Cleaved-NOTCH1, as well as the mRNA expression of Jad1 and NOTCH1. These results indicate that DOP activated the JAGGED1/NOTCH1 signaling pathway to inhibit the expression of M2-like macrophages. In other reports, it has also been confirmed that some traditional Chinese medicine and polysaccharides could achieve therapeutic effects by suppressing the NOTCH1 pathway. For example, Astragalus Polysaccharide (RAP) induced macrophage polarization to M1 via the NOTCH signaling pathway, while Ganoderic acid A inhibited ox-LDL-induced lipid deposition and macrophage inflammation in THP-1 cells via NOTCH1/PPAR gamma/CD36 signaling [49].

Previous research has indicated that the majority of TAMs amass at the tumor periphery and non-vascularized regions. Additionally, the preferential accumulation of TAMs at invasive edges has been hypothesized to significantly contribute to tumor progression [50]. Therefore, it is suggested that dynamic changes, involving chemokines and cytokines, occur in the TME. These changes lead to the promotion of monocyte recruitment and polarization into the M2 macrophage phenotypes, which contributes to tumor progression. The M1 macrophage phenotypes are suppressed as a result [51]. The NOTCH1 pathway forms a positive feedback loop between the tumor cells and TAMs, and its overexpression increases the expression of NOTCH1 and JAGGED1 and upregulates the expression of IL-1β and CCL2, thereby leading to M2 polarization [52]. In our study, DOP intervention downregulated the protein expression of Jad1, NOTCH1, and Cleaved-NOTCH1, and as well as induced the mRNA expression of IL-6, CXCL9, CXCL10, IRF1, TNF-α, STAT1, and IL-1β in the M2 phenotypes. Ultimately, CM-DOP inhibited the migration of gastric cancer cells by reversing the effects of N-cadherin and vimentin, as well as increasing the E-cadherin expression; meanwhile, CM-DOP upregulated Caspase-3 and increased the ratio of Bax/Bcl-2, thereby inducing apoptosis. These findings are consistent with previous reports on the inhibitory effects of Arsenic Trioxide (ATO) on the JAGGED1 and NOTCH1 expression, which suppressed M2 polarization through downregulating the NOTCH-dependent paracrine of CCL2 and IL1β [53]. 

## 4. Materials and Methods

### 4.1. Materials

Dendrobium officinale was purchased from Zhejiang Shouxiangu Pharmaceutical Co., Ltd. (Jinhua, China), and PMA and LPS were purchased from Sigma (Saint Louis, MI, USA). Recombinant human interleukin-4 (IL-4) was purchased from PeproTech, Inc. (Rocky Hill, NJ, USA). TNF-α and IL-6 ELISA kits were purchased from Lianke Biological Technology (Hangzhou, China). Cell Counting Kit-8 (CCK-8) Assay Kit was received from MCE (Romulus, NJ, USA). Total Protein Extraction kit was purchased from Beyotime (Shanghai, China). The MiNiBEST Universal RNA Extraction Kit, PrimeScript RT Reagent kit, and SYBR Premix Ex Taq II Kit were purchased from TaKaRa (Dalian, China). Transwell polycarbonate membrane had an 8 μm pore size (Corning City, NY, USA). The primary antibodies E-cadherin, N-cadherin, Vimentin, Caspase-3, Bax, Bcl-2, Ki67, and β-actin were purchased from Proteintech (Wuhan, China). The primary antibodies ARG1, TGM2, and Cleaved-NOTCH1 were purchased from Cell Signaling Technology (Danvers, MA, USA). The primary antibodies STAT6, p-STAT6, PPAR-r, JAGGED1, Cleaved-NOTCH1, and NOTCH1 were purchased from Abcam (Cambridge, UK). β-actin was purchased from Proteintech (Wuhan, China). The ECL Plus Western Blotting Detection Kit was purchased from Technology Co., Ltd. (Beijing, China). Anti-CD80-FITC and Anti-CD206-PE were obtained from Thermo Fisher (Waltham, MA, USA). 

### 4.2. Preparation of DOP

The extraction and purification of DOP were performed according to our previous manuscript [54], with a resulting purity of over 80%. The molecular weight of DOP was determined via high-performance gel-permeation chromatography (HPGPC). Generally speaking, the water extraction weighing 50 g was dissolved twice in 2.5 L of water and subsequently mixed with 95% alcohol at a ratio of 1:5. After a period of 24 h, the resultant mixture was collected via centrifugation at 4000 rpm for 15 min before being deproteinized via a savage reagent and dried to form crude DOP.

### 4.3. Cell Culture

The Chinese Academy of Sciences Cell Bank provided human acute monocytic leukemia cell line (THP-1) and gastric cancer cell lines (HGC27 and AGS). All cells were cultured in a RPMI-1640 medium with 10% fetal bovine serum (FBS) and supplemented with 1 mM of sodium pyruvate, 2 mM of glutamine, and 1% penicillin/streptomycin/gentamycin. The cells were set at 37 °C, 5% CO_2_. THP-1-derived macrophages were obtained with 100 ng/mL of PMA for 24 h, washed twice with PBS, and replaced with normal 1640 medium for 24 h at rest.

### 4.4. THP-1 Cell Differentiation and Treatment

THP-1 cells were seeded in 100 mm^3^ dishes at a density of 5 × 10^7^/dish. THP-1 macrophages were incubated with 20 ng/mL of IFN-γ and 100 ng/mL of LPS for 24 h to polarize to M1 subtype macrophages. The cells were co-treated with 100, 200, or 400 μg/mL DOP for an additional 24 h. To obtain the M2 subtype macrophages, the cells were then exposed to 100 ng/mL of IL-4 for 24 h and co-treated with 100, 200, or 400 μg/mL of DOP for an additional 24 h.

### 4.5. The Preparation of Conditioned Medium

Different phenotype macrophages were incubated for 24 h in a serum-free medium, and the conditioned media were named as M0 (CM-M0), M2 (CM-M2), and DOP-treated M2 macrophages (CM-H-DOP, CM-M-DOP, and CM-L-DOP), and then collected via centrifugation at 1000× *g* for 5 min and stored at −20 °C until use.

### 4.6. Cell Viability Assay

To assess the cytotoxic impact of DOP on THP-1 cells, varied doses of DOP were introduced into the culture, utilizing the CCK-8 reagent kit. THP-1 cells were seeded in 96-well plates with a concentration of 5 × 10^3^ per well for 24, 48, and 72 h. Simultaneously, HGC27 and AGS cells were seeded in a 96-well plate at a density of 3 × 10^3^ cells/well and then cultured for 24 h in various CMs supplemented with 5% FBS. Afterward, 10 μL of the CCK-8 solution was added to each well, and the plate was then incubated at 37 °C for a duration of 4 h. The optical density was recorded at 450 nm in every well using a microplate ELISA reader (Bio-Rad Laboratories, Hercules, CA, USA). The calculation of relative cell viability was conducted using the following equation: relative absorbance = (mean absorbance at each time point/mean respective absorbance).

### 4.7. Measurement of TNF-α and IL-6 via ELISA

After treatment with various concentrations of DOP, we collected the supernatants to evaluate the secretion of TNF-α and IL-6. The measurement was carried out using ELISA kits according to the manufacturer’s instructions.

### 4.8. Phenotype Analysis via Flow Cytometry

Briefly, macrophage cells were stimulated via IL-4 and DOP, harvested immediately in cold PBS containing 2% FBS, and washed with staining buffer twice. Next, the suspension was stained with a 5 μL anti-CD80-FITC and anti-CD206-PE antibody for 30 min and washed twice. Fluorescence was measured via flow cytometry after gating on FSC/SSC parameters using a MoFlo XDP (Beckman Coulter, Pasadena, CA, USA) and then analyzed using FlowJo^®^ VX software 7.6.2 (Tree Star, Inc., Ashland, OR, USA).

### 4.9. Real-Time PCR

Total RNA was extracted using RNA extraction reagent kits following the manufacturer’s protocol. Reverse transcription was performed using a PrimeScript RT Reagent kit with 1 μg of total RNA. The cDNA corresponding to the same amount of RNA was taken and detected via a Light Cycler 480 II PCR Detection system (Roche, Indianapolis, IN, USA). The reaction system, consisting of the cDNA, forward, and reverse primers, and the SYBR Green PCR master mix, was 25 μL. Analysis of all data was performed using the β-actin gene expression as an internal reference. The specific primers are listed in Table 1. For each experiment, we tested multiple reference genes to ensure no effects were due to treatment. Following this, we adjusted the gene expression data using the 2^−ΔΔCT^ technique with the appropriate reference gene [55].

After the M0 macrophages were induced via PMA to adhere to the well for 24 h, the cellular medium was aspirated and washed three times with PBS. Simultaneously, IL4 and DOP drugs were added. After the DOP treatment for 24 h, the cells were collected and the total proteins were extracted. Then, the sample underwent SDS-polyacrylamide gel electrophoresis and was then transferred to a polyvinylidene fluoride (PVDF) membrane via the wet transfer method. Following this, the membrane was covered with 5% skim milk for 1 h and left to incubate with the primary antibody at 4 °C overnight. The protein expression was determined using a horseradish-peroxidase-conjugated antibody, followed by an enhanced chemiluminescence assay. An image analysis scanning system was used to collect the intensity of the bands. β-actin served as the internal control.

### 4.10. Effect of CM-DOP on Gastric Cancer Cell

Transwell polycarbonate membrane (8 μm pore size) was incubated with the membrane at 37 °C for 6 h. HGC-27 gastric cancer cells were trypsinized and resuspended in RPMI1640 containing 10% FBS at a density of 1 × 10^6^ cells/mL. Cell suspensions (100 μL) were seeded in the upper chamber at a density of 2 × 10^5^/well, and the lower chambers were added to a conditioned medium separately (CM-M0, CM-M2, CM-H-DOP, CM-M-DOP, and CM-L-DOP). After 24 h of invasion, the cells migrated to the bottom of the filter membrane. Then, we stained the membranes with 1% methylrosanilinium chloride and randomly selected five visual fields for counting using an optical microscope. Additionally, HGC-27 and AGS gastric cancer cells were adhered to 100 mm^3^ dishes for 24 h, the cellular medium was aspirated and washed three times with PBS, and then the conditioned media (CM-M0, CM-M2, CM-H-DOP, CM-M-DOP, and CM-L-DOP) were added to the dishes separately. After 24 h, the cells were collected, and the protein was extracted for WB detection.

### 4.11. In Vivo Assay

Specific pathogen-free male BALB/c nude mice (6–8 weeks old) were obtained from Vital River Laboratory Animal Technology Co., Ltd., Beijing, China (Certification No. SGXK(Jing)2016-0011). The Guide for the Care and Use of Laboratory Animals was followed in all animal experiments. The animals were reared under pathogen-free conditions (temperature 20 ± 2 °C, 12/12 h light–dark cycle) and allowed to eat and drink freely. The experimental animals were divided into three groups at random: Ctrl, H-DOP (3 g/kg/day), and L-DOP (1.5 g/kg/day; n = 6 per group). Then, 1 × 10^7^ of HGC-27 cells were subcutaneously implanted into the right axillary of mice. DOP was administered via gavage daily for 24 days, starting from day 7 after injection. After 24 days, the mice were euthanized, and their tumors were collected, dissected, and weighed. Fresh tumors were used for immunohistochemical analysis. The slides underwent an overnight incubation with the primary antibodies at 4 °C and were subsequently subjected to incubation with fluorescent secondary antibodies. Finally, the slides were observed via fluorescence microscopy, and images were captured from five different views.

### 4.12. Statistical Analysis

Statistical analysis was conducted using GraphPad Prism 8 software (San Diego, CA, USA). One-way ANOVA was performed to distinguish multiple comparisons between the experimental groups. The data are presented as the mean ± standard deviation (SD). Any *p*-values of <0.05 were deemed to be statistically significant.

## 5. Conclusions

The DOP treatment regulated the polarization of IL-4-induced M2 macrophages into M1 phenotypes; decreased the mRNA levels of M2 markers Arg1, TGM2, Fizz1, Alox15, and PPARr; and increased the mRNA expression of IL-6, CXCL9, CXCL10, TNF-α, and IL-1β in the M2 subtype macrophages. These results suggest that DOP has the potential to polarize the phenotype of THP-1 macrophages from M2 to M1. Treatment with DOP inhibited the STAT6/PPAR-r and JAGGED1/NOTCH1 signaling pathways in M2 subtype macrophages and reduced the protein expression of ARG1 and TGM2. CM-DOP inhibited migration by reducing the expression of N-cadherin and vimentin, increasing E-cadherin, and promoting apoptosis through upregulating Caspase-3 and increasing the ratio of Bax/Bcl-2 on gastric cancer cells. These results highlight that DOP may inhibit gastric cancer by regulating macrophage polarization from the M2 into the M1 subtype, thereby reducing migration and inducing pro-apoptosis. Our findings suggest that, for the prevention and treatment of gastric tumors, DOP may be a therapeutic option.

## Figures and Tables

**Table 1:** RT-PCR primers used in this experiment.

Gene	Forward Primer (5′ to 3′)	Reverse Primer (5′ to 3′)
*IL-6*	TACATCCTCGACGGCATCTC	CCATCTTTGGAAGGTTCAGG
*Fizz1*	GCAAGAAGCTCTCGTGTGCTAGTG	CGAACCACAGCCATAGCCACAAG
*ARG 1*	CACACCAGCTACTGGCACACC	GCAACTGCTGTGTTCACTGTTCG
*TGM2*	GCAGTGACTTTGACGTCTTTGCCC	GTAGCTGTTGATAACTGGCTCCACG
*CXCL9*	GCTGGTTCTGATTGGAGTGC	GAAGGGCTTGGGGCAAATTG
*CXCL10*	CCTTATCTTTCTGACTCTAAGTGG	CTAAAGACCTTGGATTAACAGG
*IRF1*	GGAAGGGAAATTACCTGAGG	CTCCAGGTTCATTGAGTAGG
*ALOX15*	CAGATGTCCATCACTTGGCAG	CTCCTCCCTGAACTTCTTCAG
*STAT1*	CAATGCTTGCTTGGATCAGC	GTGATAGGGTCATGTTGCTAGG
*IL1β*	AATGATGGCTTATTACAGTGGCA	GTCGGAGATTCGTAGCTGGA
*PPAR-r*	TTCAGAAATGCCTTGCAGTG	CCAACAGCTTCTCCTTCTCG
*TNF-a*	GGAGAAGGGTGACCGACTC	TGGGAAGGTTGGATGTTCGT
*NOTCH1*	GAGGCGTGGCAGACTATGC	CTTGTACTCCGTCAGCGTGA
*JAGGDE1*	TGACCAGAATGGCAACAAAA	GTTGGGTCCTGAATACCCCT
*β-actin*	GTGGCCGAGGACTTTGATTG	CCTGTAACAACGCATCTCATATT

## Data Availability

Most of the data utilized in drafting this manuscript can be found in the Results sections. For additional information regarding the methods and raw files, please contact the corresponding authors.
